# Clinical learning of nursing students during the COVID-19 pandemic in Limpopo province, South Africa

**DOI:** 10.4102/curationis.v47i1.2578

**Published:** 2024-09-23

**Authors:** Linda Nchabeleng, Mamare A. Bopape, Ledile E. Manamela, Tshepo A. Ntho

**Affiliations:** 1 Department of Nursing Science, Faculty of Health Sciences, University of Limpopo, Polokwane, South Africa

**Keywords:** clinical learning, experiences, nursing students, COVID-19, pandemic, nursing education institution

## Abstract

**Background:**

The Nursing Education Programme was affected during the coronavirus disease 2019 (COVID-19) pandemic, resulting in nursing students being unable to participate in the clinical experiential learning required by the South African Nursing Council.

**Objectives:**

The study seeks to explore and describe nursing students’ experiences of clinical experiential learning during the COVID-19 pandemic.

**Method:**

A qualitative, explorative and descriptive, research design was used in the study. A non-probability purposive sampling method was used, and 55 nursing students participated in the study. Data were collected through six focus group discussions, consisting of 8–12 nursing students in each group. Data were analysed following Tesch’s open coding method.

**Results:**

Three themes emerged from the study’s findings: The impact of COVID-19 on the clinical experiential learning of nursing students, the effects of COVID-19 on the mental well-being of nursing students, and nursing students’ experiences of support during the COVID-19 pandemic. Notably, 11 sub-themes emerged.

**Conclusion:**

The findings of this study reveal that the COVID-19 pandemic severely disrupted the Nursing Education Programme, highlighting the challenges of inadequate clinical hours, restricted clinical access and the significant psychological impact on students.

**Contribution:**

This study adds to the literature on students’ experiences during clinical experiential learning in South Africa during the COVID-19 pandemic.

## Introduction

The World Health Organization (WHO) declared a Public Health Emergency of International Concern in January 2020 and later classified the coronavirus disease 2019 (COVID-19) as a pandemic in March 2020 (WHO 2022). On 15 March 2020, President Cyril Ramaphosa proclaimed a national disaster in South Africa in response to the WHO’s report on the COVID-19 pandemic (Motala & Menon 2020). Educational institutions were temporarily closed to control the spread of the COVID-19 pandemic (Fowler et al. 2021). Globally, many higher education institutions (HEIs) shut down campuses or switched from on-campus to online learning; students were not exempted from this disruption (Hung et al. 2021). Based on the WHO recommendation, the South African government agreed to shut down educational institutions to contain the spread of the COVID-19 pandemic (Alex 2022). The early closure of the HEIs meant that they had to develop a contingency plan.

The education industry underwent significant changes because of the closure of educational institution (Babbar & Gupta 2022). The changes included the transition from traditional pedagogy to online teaching and learning modes, which has significantly affected students’ clinical experiential learning. International studies have found that the COVID-19 pandemic increased stress levels in nursing professionals and trainee nurses (Dziurka et al. 2022; Godbold et al. 2021; Velarde-García et al. 2021). Furthermore, Aslan and Pekince (2020) confirm that COVID-19 infections in Turkey raised the stress levels of healthcare personnel, particularly nursing students. Moreover, in Africa, the planned academic activities for nursing and midwifery students were postponed during the COVID-19 pandemic because the healthcare system struggled to provide adequate personal protective equipment (Hugo-Van Dyk et al. 2022). The shift also impacted the Nursing Education Programme, as nursing students had to withdraw from clinical experiential learning. The literature review further finds that nursing students were confronted with other challenges in the clinical areas during the COVID-19 pandemic. These challenges include psychological distress like depression and anxiety, stress, lack of clinical exposure and experiential hours, future employment uncertainty, the effect of delayed study completion and a lack of support (Alcalá-Albert et al. 2022; Mpasa et al. 2021; Susmarini et al. 2022).

In South Africa, nursing education programmes and students were also affected by the disruption of the COVID-19 pandemic. For example, a study conducted by Molefe and Mabunda (2022) among students at a public nursing college in South Africa reveals challenges such as a lack of clinical exposure and an inability to meet the required clinical hours. Despite all these challenges, nursing students had to comply with all placement requirements of the nursing education programmes, prescribed by the South African Nursing Council (*Nursing Act 2005*). These include students accumulating a total of at least 2800 clinical learning hours; this is in accordance with Education and Training Regulation 174 of the South African Nursing Council (SANC 2013, n.d.). Regulation 174 leads to their registration as professional nurses and midwives. Significantly, practical training should make up 60% of the total duration of the course. However, during the COVID-19 pandemic, nursing students faced challenges in accumulating the minimum number of clinical practice hours. This implies that the clinical experiential learning of nursing students was compromised, affecting their clinical practice and competence (Aslan & Pekince 2020). Of concern is that the incompetence of the nursing students will spill over to future practice, leading to a decline in the quality of healthcare services. Thus, healthcare services are unlikely to achieve Sustainable Development Goal (SDG) 3, which strives for healthy lives and well-being for all ages (United Nations 2015).

This study documents nursing students’ experiences regarding clinical experiential learning during the COVID-19 pandemic in a selected National Education Institute (NEI) in Limpopo province. The findings of this study could significantly enhance the use of evidence in policy formulation and decision-making to create flexible educational frameworks that will promote effective learning and mental well-being of nursing students, especially during global health crises. Furthermore, the study’s findings may assist in planning and developing effective interventions to achieve the African Union Agenda 2063, which seeks to improve the health of all citizens and initiate a skills revolution underpinned by science, technology and innovation.

### Aim

The researcher seeks to explore and describe the experiences of nursing students at the selected NEI, regarding their clinical placement during the COVID-19 pandemic.

## Research methods and design

A qualitative research design was employed to explore and describe nursing students’ experiences regarding clinical placement during the COVID-19 pandemic. Explorative and descriptive research designs were used to gain greater insight into the experiences of the nursing students of the selected NEI regarding clinical placement during the COVID-19 pandemic. To get the participants’ viewpoints, a descriptive design was used to produce data that describe the who, what and where of their events or experiences (Doyle et al. 2020).

### Research setting

The study is conducted at an NEI in the Limpopo province of South Africa. This NEI is located in the Capricorn District, 32 km from Polokwane. It is one of the eight historically disadvantaged HEIs in South Africa. South African Nursing Council has accredited the NEI to train nursing students under Regulation 174 (SANC 2013). The selected NEI provides a 4-year Bachelor of Nursing Science programme and advanced postgraduate degrees, such as Masters degrees and Doctor of Philosophy in nursing. For the academic year 2021, a total of 284 nursing students were enrolled in the R174 programme. The programme encompasses both theoretical and clinical experiential learning, with classroom-based instruction for theory and attendance at accredited clinical institutions for clinical experiential learning which enables nursing students to integrate theory into practice.

### Population and sampling

The population included all 284 nursing students who were enrolled on the Bachelor of Nursing Science degree programme for the academic year 2021 at the selected NEI. In addition, a non-probability purposive sampling method was used to invite 19 undergraduate nursing students from the second year, 16 from the third year and 20 from the fourth year. In total, 55 undergraduate nursing students participated in the study, based on their experiences of clinical placement during the COVID-19 pandemic; six focus group discussions (FGDs) were conducted, each comprising eight or nine nursing students. The study did not include first-year nursing students who registered in 2022 because the data were collected from January to April 2022. During this period, students were busy with theory in the classroom and had not yet been placed for clinical experiential learning.

### Data collection

The primary researcher collected data through face-to-face semi-structured FGDs over a period of 3 months. Focus group discussion is a data collection method in which participants engage in diverse activities such as discussion, inquiry and sharing opinions and experiences related to research questions in a group situation (Andarmoyo 2022). In this study, nursing students were recruited with the help of class representatives, using flyers posted on notice boards and shared in WhatsApp groups. The researcher emphasised to the class representatives that their role was to disseminate recruitment flyers in the WhatsApp group and not to pressure or coerce potential participants into joining the study. Those interested in participating in FGDs were asked to send a message on WhatsApp, after which the primary author followed up with them. The primary author, who collected the data, had no relationship with nursing students, which might have pressurised students to participate in the study. Only the nursing students who signed the informed consent form participated in the study. This study consists of six FGDs, with 8–12 nursing students in each group. The researcher asked the participants the following central question, ‘Can you please describe your experiences regarding clinical placement during the COVID-19 pandemic?’ Nursing students who participated in this study were encouraged to speak freely and to share their experiences. During FGDs, an audio recording device was used, with the consent of the participants, to capture the discussion between the participants. In addition, field notes were taken to document non-verbal cues exhibited by the participants. The primary author used an interview guide that underwent a pilot FGD study with a group of eight students. Students who participated in the pilot study were excluded from the main study. The pilot study’s findings assisted the researcher in refining the interview guide, using probing skills and managing group dynamics. The FGDs were conducted in the skills laboratory boardroom, which was free from noise. A notice indicating that an interview was in progress was pasted on the main door to avoid interruptions. The FGDs were conducted in English, as it is the medium of instruction of the NEI, and they lasted for 45 min to 60 min. Data saturation was reached after the fifth FGD (Hennink & Kaiser 2022). However, to ensure and confirm that no new information emerged, the primary researcher continued data collection for one more FGD.

### Data analysis

Tesch’s eight-step inductive, descriptive and open-coding technique (Creswell 2014) was used to analyse the collected data. The researcher thoroughly reviewed data after each FGD to gain a comprehensive understanding, which provided essential background and contextual information. The researchers picked one interview to read and tried to make sense of the content by jotting down any thoughts that came to mind. Subsequently, in step 3, the researchers arranged related subjects into columns, labelled topics, unique topics and leftovers. In step 4, the researcher then abbreviated the topics as coded and wrote codes next to the appropriate segment of the text. The researchers then examined the organisation of the data to check whether new categories or codes had emerged. The most descriptive language for the themes was found and converted into categories by the researchers in step 5. In step 6, the abbreviation of each category was decided, and the codes were listed alphabetically. The researcher collected all the data associated with each theme and consolidated it into one column for analysis in step 7. Afterwards, the researcher and an independent coder, an experienced qualitative researcher with a PhD in Nursing Science, convened with the researchers to discuss and agree on the themes and sub-themes that each of them had independently identified. Lastly, in step 8, there was no need for recoding.

### Measures to ensure trustworthiness

Trustworthiness in this study was guaranteed following the four components namely, credibility, transferability, confirmability and dependability as shown in Table 1 (Mphasha, Mothiba & Skaal 2023; Ramathuba & Ndou 2020).

**TABLE 1:** Measures of trustworthiness.

Strategy	Criteria	Applicability
Credibility	Prolonged engagement	The researcher conducted FGDs with nursing students lasting 45 min to 60 min. Probing was utilised to gather detailed information.
	Multiple investigators	The supervisors were engaged throughout the research process, from conceptualisation to the final report, providing valuable input and feedback.
	Peer-review	The research underwent review by experienced qualitative research supervisors in the faculty, and was presented at the Nursing Department Research Symposium.
	Member checking	After each interview, the researcher summarised the information and asked the nursing students participating in the FGD to validate its accuracy.
Transferability	Data saturation	FGD-5 yielded no new data. However, one additional FGD was conducted to confirm data saturation. No new information was obtained, and thus data collection was stopped.
	Thick description	The study’s setting, participants, sampling procedure and methodology were thoroughly explained, thus enabling other researchers to apply the findings.
Confirmability	Audit trail	The supervisor thoroughly analysed and verified the data to ensure consistency of the themes and sub-themes. Afterwards, the verbatim transcripts, audio recordings and field notes were given to an independent coder. The coder’s findings were compared with the original analysis to identify similarities or differences, and a consensus was reached.
Dependability	Dense description of research methods	The research methodology employed in this study is thoroughly and accurately described and underwent rigorous peer-reviews by respected faculty researchers.

*Source:* Adapted from Mphasha, Mothiba and Skaal (2023) and Ramathuba and Ndou (2020).

Note: Please see full reference list of this article Nchabeleng, L., Bopape, M.A., Manamela, L.E. & Ntho, T.A., 2024, ‘Clinical learning of nursing students during the COVID-19 pandemic in Limpopo province, South Africa’, *Curationis* 47(1), a2578. https://doi.org/10.4102/curationis.v47i1.2578 for more information.

FGD, focus group discussions.

### Ethical considerations

Before conducting the study, ethical approval was obtained from the University of Limpopo, Turfloop Research and Ethics Committee (TREC) (Project Number: TREC/358/2022: IR). Permission to conduct the study was sought and granted by authorities in the selected NEI in the Limpopo province. Before participating in the study, each nursing student in an FGD was provided with an information leaflet about the study. They were then allowed to ask questions before signing. In addition, the researcher informed participants that they had the right not to participate in the study or that they could leave at any moment with no repercussions. The researcher ensured the protection of information obtained from the participants, in line with the *Protection of Personal Information Act (POPIA) No. 04 of 2013*. The researcher took great care to safeguard the anonymity of all nursing students who participated in the study, ensuring that their identities remained confidential and undisclosed to anyone outside the research team. To achieve this, pseudonyms were assigned to each participant during data collection, with nursing students being referred to as Participants 1, 2 or 3 during the FGDs. To address the potential risk of emotional and psychological harm that might have arisen among nursing students during FGDs when they shared their clinical learning experiences, the primary author, who is a registered nurse, implemented several precautionary measures during FGDs. These measures included moderating the discussions, conducting debriefing sessions after each FGD and ensuring that institutional psychological support services were readily available for any unexpected emotions that might have arisen.

## Results

The results of this study are presented according to the demographic data and include themes and sub-themes.

### Characteristics of the nursing students

A total of 55 students were involved, 9 male and 46 female. Based on their level of studies, 19 nursing students were in their second year, 16 were in their third year and 20 were in their final year. The following are participant codes used, e.g., Participant (1–9), focus group (D, E, F, G, H & I), Female or Male (F or M) and the level (2, 3 or 4) (see Online Appendix 1).

### Themes and sub-themes

Three themes emerged, namely: The impact of COVID-19 on the clinical experiential learning of nursing students, the effects of COVID-19 on the mental well-being of nursing students, and nursing students’ experiences of support during the COVID-19 pandemic. Notably, 11 sub-themes emerged. Direct quotations from nursing students are captured in ‘*italic format’* to support the research findings. Table 2 shows the themes and sub-themes that emerged from the findings.

**TABLE 2:** Themes and sub-themes that emerged from the data analysis.

Themes	Sub-themes
1. Impact of COVID-19 on clinical placement of nursing students	1.1. Incompetence in the performance of some skills 1.2. Compromised integration of theory and practice 1.3. Inability to meet the clinical hours required by the South African Nursing Council 1.4. Unfair and inappropriate utilisation of nursing students during clinical placement 1.5. Restricted exposure to the clinical areas during the hard lockdown
2. Effects of COVID-19 on the mental well-being of nursing students	2.1. Fear of COVID-19 infection 2.2. Fear of patients 2.3. Fear of death 2.4. Feeling of depression and desperation
3. Nursing students’ experiences of support during the COVID-19 pandemic	3.1. Inadequate support from the lecturers and supervisors 3.2. Inadequate supervision by the senior personnel in the clinical areas

COVID-19, coronavirus disease 2019.

#### Theme 1: Impact of COVID-19 on clinical placement of nursing students

The FGDs revealed that COVID-19 had a significant impact on the clinical placement of nursing students. The following sub-themes emerged: incompetence in the performance of some skills, compromised correlation of theory and practice, inability to meet the clinical hours required by SANC, unfair and inappropriate utilisation of nursing students during clinical placements, and restricted exposure to clinical areas during the hard lockdown.

**Sub-theme 1.1: Incompetence in the performance of some skills:** The findings indicate that the nursing students found it difficult to perform tasks they had not previously performed and that they were not familiar with the equipment being used for reasons relating to lockdown regulations. These findings are supported by the following participant statements:

‘As for me, I was not okay throughout. We returned around the end of the year after the lockdown and had to go to practicals. The nurses were putting pressure on us and expected us to know some of the skills that we did not practice because of the lockdown. It was too much, and they expected you to know.’ (Participant 9E, F, Level 2)

‘Uhm, it was a little bit bad, but we had to ask the sisters at the hospital what the equipment was used for and what the procedure was. How is it done so.’ (Participant 5I, F, Level 4)

**Sub-theme 1.2: Compromised integration of theory and practice:** The findings reveal that nursing students had trouble putting theory into practice during lockdown procedures, which led to deficient performance of clinical skills and an inability to fulfil the learning objectives. These findings are confirmed by the following participant statements:

‘For theory, we were learning online, but for clinicals, we were not going anywhere. So, for theory, we had information, but for clinicals, some skills, like CPR, were taught online. But doing it in the hospital was hectic.’ (Participant 1F, F, Level 4)

‘As for my learning, during COVID-19, we had to learn online. Although we were able to cover the theoretical aspects of our courses, the problem was that we couldn’t apply what we learned practically due to the restrictions. This meant that we couldn’t see the conditions we were studying in real life, making it difficult to fully comprehend and retain the knowledge we gained through online learning.’ (Participant 6F, F, Level 4)

**Sub-theme 1.3: Inability to meet the clinical hours required by the South African Nursing Council:** The nursing students reported a shortfall in their clinical hours because of the COVID-19 restrictions and lockdown restrictions; this affected their ability to access clinical areas. This sub-theme is affirmed by the following statement from one of the participants:

‘[*M*]y main problem during my clinical placement was that we were divided into groups, and each group was assigned a specific week to attend the clinical area. As a result, I was worried that I might miss out on some hours of clinical training if I missed a few days.’ (Participant 8F, M, Level 4)

Another participant added that student nurses could not go into the hospitals without first being vaccinated for COVID-19:

‘Regarding the clinical placement, it was hard for us because sometimes, at the hospital, they would tell us we should not come to the hospital without being vaccinated.’ (Participant 7G, F, Level 2)

**Sub-theme 1.4: Unfair and inappropriate utilisation of nursing students during clinical placements:** The nursing students indicated that they performed tasks unrelated to nursing and beyond their scope of practice while at clinical sites during the COVID-19 pandemic. This did not align with their intended learning objectives. This was corroborated by the following statement of one of the participants:

‘[*B*]ecause we were being sent on errands, on personal errands which are not in our scope and that was exposing us to contacting the COVID-19 disease more because we were going around the hospital.’ (Participant 7E, F, Level 2)

Another participant further stated that:

‘[*S*]isters used to send us around like porters. We were tasked with taking patients from orthopaedic to x-ray, and then when we returned, they would ask us to go and get the patient from wherever they had been moved to. We would then take the patient to orthopaedic, and from there, we would be asked to go and get medication from the pharmacy. After that, we would have to go to the mortuary, and at the end of the day, we would not have learned anything new. We were just messengers for the day.’ (Participant 1I, M, Level 4)

**Sub-theme 1.5: Restricted exposure to clinical areas during hard lockdown:** The research findings show that during the stringent lockdown, nursing students had limited access to clinical areas because they were required to stay at home. At the beginning of the lockdown, their understanding of COVID-19 was limited Participants stated:

‘[*D*]uring COVID-19, the hospitals that we were allocated didn’t allow students to go because they said that we didn’t know anything about COVID-19 and that if we ever contracted it, it would be a problem. So, I cannot say I have working experience at the hospital during COVID-19 because I wasn’t placed.’ (Participant 1I, M, Level 4)

‘[*I*]t’s not like we didn’t want to go to a hospital, we were forced to stay at home due to the pandemic.’ (Participant 11I, M, Level 4)

#### Theme 2. Effects of COVID-19 on the mental well-being of nursing students

The mental well-being of the student nurses was impacted by the difficulties they faced in clinical settings. These included fear of COVID-19 infection, fear of patients, fear of death, and feelings of depression and desperation.

**Sub-theme 2.1: Fear of COVID-19 infection:** The study findings reveal that the nursing students were anxious about and afraid of contracting COVID-19 while in the clinical areas. Sometimes they even felt that they had symptoms of the infection. This finding is corroborated by the following participant statements:

‘So, we became scared because we thought that we were going get COVID-19, and sometimes we became delusional and think like we have those symptoms for a little, so it was scary for me.’ (Participant 3F, M, Level 4)

‘It was just too much, especially working in clinical units like casualty, where you have most patients who present with COVID-19 symptoms. You come back being anxious that you might have contracted the disease.’ (Participant 5E, M, Level 2)

**Sub-theme 2.2: Fear of patients:** The narratives indicate that the participants were afraid of interacting with patients and delivering patient care at clinical placements during the COVID-19 pandemic. This is affirmed by the following statements:

‘[*E*]hh, my experience was quite scary and uncomfortable because we would go there, and we would even fear touching the patients. I remember this other time a patient asked me to take out his shoes, and then I thought of COVID-19 because his file said that he was COVID-19 positive.’ (Participant 2D, F, Level 3)

‘[*H*]onestly, coming back to practicals was not easy. I was anxious and I was always like I did not want to treat any patient because you do not know which one has COVID-19. Based on the symptoms, I was thinking maybe everyone has COVID-19, and I did not know how to treat those patients.’ (Participant 4E, F, Level 2)

**Sub-theme 2.3: Fear of death:** The findings reveal that the nursing students were fearful of catching the COVID-19 virus and dying after providing care to patients who had tested positive for the virus. This finding is endorsed by the following quotation from one of the participants:

‘Sometimes, we would go inside the cubicle, and after leaving the cubicle, we would find out that the patient we were helping was COVID-19 positive. That affected us psychologically, and we were stressed because we feared that we might die.’ (Participant 3D, F, Level 3)

Another participant ratified this statement stating:

‘[*S*]o, during the COVID-19 pandemic, to be genuinely speaking, I was terrified because at that time, like most healthcare providers, we were dying, so when we were going to the practical, we were getting scared. I was getting scared that I might contract the disease and die from the pandemic.’ (Participant 3E, F, Level 2)

**Sub-theme 2.4: Feeling of depression and desperation:** According to the study’s findings, some of the nursing students felt depressed and even desperate during the COVID-19 pandemic. This finding is verified by the following statement:

‘[*M*]y mental state was unstable. At times, you felt depressed and anxious, and panic attacks were expected. And having to go to clinical practice during that era was not easy because everyone is dying.’ (Participant 8H, F, Level 3)

Another participant added:

‘Uhh, for me, it was psychologically challenging, especially when I returned from my practicals and saw news headlines showing the increasing number of cases and thinking that it might be me tomorrow.’ (Participant 2I, F, Level 4)

#### Theme 3. Nursing students’ experiences of support during the COVID-19 pandemic

The nursing students reported a lack of support from lecturers and inadequate supervision from senior personnel in the clinical settings during the COVID-19 pandemic.

**Sub-theme 3.1: Inadequate support from the lecturers and supervisors:** The study findings indicate that the participants experienced a lack of support from their teachers and supervisors, and that they had no one to support them when challenges arose at the clinical placements during the COVID-19 pandemic. This is verified as follows:

‘Uhm, I remember this other time when we contacted our lecturers and told them that we had been in contact with Corona patients, and they did nothing about that.’ (Participant 1D, F, Level 3)

‘… I also remember that I was once quarantined, but I did not mention that to my lecturers because they would not do anything; they would even force me to replace hours, while I was not the one who just went outside and said I wanted COVID-19.’ (Participant 4D, F, Level 3)

**Sub-theme 3.2: Inadequate supervision by the senior personnel in the clinical areas:** The nursing students in the study maintained that they had inadequate supervision from the senior personnel, who were also afraid of the COVID-19 virus and sent the student nurses to carry out clinical skills alone at the clinical placements. The following statements support this finding:

‘[*U*]hm my experience was very scary, you would find sometimes the sisters at the hospital, we were all scared so instead of them going with us, supervising us during the skills they will just send us alone there.’ (Participant 4D, F, Level 3)

Another participant also alluded that:

‘[*O*]kay, if I remember correctly, my clinical placement was very scary since we were doing the first year in 2020 and did not know anything. When we needed the nurses, the “in-charges”, the matrons and the professional nurses, they were also scared, so they mainly sent us to do stuff, especially when they saw that the patient was showing signs of COVID-19 they would send us and leave us alone with those patients.’ (Participant 8D, F, Level 3)

## Discussion

Nursing students’ education and their acquisition of clinical competence depend profoundly on their clinical learning experience (Muthelo et al. 2023; Nordquist et al. 2019; Ntho 2020). The findings of the current study reveal that nursing students found it difficult to practise some of the necessary skills during their clinical experiential learning. Because of the temporary closure of the NEIs to limit the spread of the COVID-19 pandemic, teachers and supervisors could not demonstrate their clinical skills in the simulation laboratory. The findings of this study are congruent with those of the review conducted by Molato and Sehularo (2022), which confirm that the demonstration of clinical skills in a simulation laboratory was not possible during the COVID-19 pandemic. The participants in this study report that they found it challenging to integrate theory into practice in clinical areas during the COVID-19 pandemic. Similarly, Mpasa et al. (2021) found that nursing students feared they might fail to meet their required learning objectives during the COVID-19 pandemic.

The study findings further show that the COVID-19 regulations and the inability to access clinical areas during the hard lockdown led to the nursing students not meeting the required number of clinical hours. Moreover, the study conducted by Molefe and Mabunda (2022) highlights that achieving the training hours required by the SANC was challenging. In addition, a study conducted in Indonesia also discovered that, because of the regulations of the clinical institutions, clinical attendance time was shortened, resulting in inadequate clinical hours for nursing students (Susmarini et al. 2022). This implies that nursing students had reduced clinical experiential learning opportunities during the pandemic and, as a result, they were not adequately prepared for future practice. Thus, Molato and Sehularo (2022) argue that there is an urgent need for the current traditional nursing education curriculum to be redesigned to prepare adequately for future pandemics.

The nursing students further reported that they had to engage in non-nursing duties, unrelated to their learning outcomes and outside their scope of practice when they were in the clinical areas during the COVID-19 pandemic. The results of another study affirm this, where the nursing students complained that instead of doing their nursing duties during the COVID-19 pandemic, they were carrying out non-nursing responsibilities, like opening emergency files and transferring laundry and equipment (Sperling 2021). Nursing students should be able to accomplish their clinical experiential learning goals and outcomes in a supportive clinical experiential learning environment.

In addition, the study demonstrates the effects of the COVID-19 pandemic on the mental well-being of nursing students. In essence, the nursing students voiced feeling anxious and scared about contracting COVID-19 in the clinical areas. The study findings are in accordance with other studies that found that nursing students were afraid of contracting the virus and spreading it to their families and patients (Dziurka et al. 2022; Susmarini et al. 2022; Zhu, Wang & Wang 2021). During the COVID-19 pandemic, nursing students were affected by psychological distress. Some participants expressed their fear of death and shared that they felt depressed and desperate in the clinical areas. These results are ratified by the study conducted by Kealeboga, Ntsayagae and Tsima (2022), who also found that the deaths of patients and other nursing staff members exacerbated the nurses’ fear of dying during the COVID-19 pandemic. The findings of this study reveal that nursing students felt depressed and desperate during the COVID-19 pandemic in clinical practice. Literature examination reveals that nurses including nursing students were confronted with psychological distress, including depression, and had to take antidepressants (Eweida et al. 2020; Kealeboga et al. 2022). Concurring with the above findings, the systematic review by Mulyadi et al. (2021) discovered that, during the COVID-19 pandemic, roughly half (50.4%) of nursing students developed depression. The COVID-19 pandemic significantly impacted nursing students’ mental well-being, negatively affecting their clinical experiential learning.

The lecturers and supervisors in this study were reported to be unsupportive; this further hampered the nursing students’ experiences in the clinical areas. They felt that they had no one to support them when challenges arose in the clinical placements during the COVID-19 pandemic. The findings of the study conducted in another NEI in South Africa find that supervisors’ roles in supporting nursing students during the COVID-19 pandemic were hindered because of their fear of contracting the virus (Hugo-Van Dyk et al. 2022). From the above discussion, it is concerning that the lecturers and supervisors did not support nursing students during the COVID-19 pandemic. Receiving direct assistance and support from preceptors might have assisted nursing students in coping in the clinical areas. The nursing students in the study indicated that they had inadequate supervision from the senior personnel, who were also afraid of the COVID-19 virus and would send them to perform the clinical skills alone in the areas of clinical placement during the COVID-19 pandemic. The above findings align with other studies carried out during the COVID-19 pandemic, where nursing students were found to be treated unfairly and the chief nurses frequently left the nursing students unattended and unsupervised (Alcalá-Albert et al. 2022; Dziurka et al. 2022). Furthermore, Ulenaers et al. (2021) recommend that support from nursing students’ lecturers and supervisors is essential to prevent a negative impact on nursing students’ learning outcomes.

### Limitations

The study takes place at the University of Limpopo in the Limpopo province, South Africa. Data were collected from 2nd, 3rd and 4th-year undergraduate students enrolled at the University of Limpopo to study the Bachelor of Nursing degree and first-year nursing students were excluded. Because of the limited reach of the study and the exclusion of 1st-year students, the results cannot be generalised to other nursing institutions in the Republic of South Africa.

### Recommendations

It is recommended that NEIs have a robust infrastructure that accommodates nursing students’ clinical experiential learning needs during health crises like the COVID-19 pandemic. This infrastructure should include a state-of-the-art simulation laboratory, adequately equipped and spacious enough to accommodate all nursing students.

It is also recommended that NEIs provide comprehensive psychological support services, including free stress and anxiety management and debriefing sessions for students. These are essential for maintaining the mental well-being of students who face challenges like the pandemic.

In a collaborative effort, it is recommended that NEIs and policymakers develop and enforce strategies that ensure the continuity of clinical placements for nursing students during pandemics. These should include creating contingency plans that can be quickly deployed to adjust to changing health crisis scenarios, thus guaranteeing that students’ practical training and professional preparedness are not compromised.

Furthermore, it is strongly recommended that NEIs ensure the availability of adequate preceptors or clinical accompanists to support nursing students in their clinical experiential learning.

Lastly, future research should be conducted to develop guidelines and evidence-based recommendations that can be implemented and can address the challenges nursing students face during clinical experiential learning amid future pandemics.

## Conclusion

Clinical experiential learning is a cornerstone of nursing education, offering students experiential opportunities to acquire clinical skills and competence. This study uncovers various challenges that nursing students encountered in the clinical areas during the COVID-19 pandemic. These challenges include insufficient clinical hours, restricted clinical access and significant psychological impacts on students. These findings highlight the urgent need for the implementation of resilient, flexible educational strategies in nursing education programmes to ensure continuity and quality of clinical experiential learning amid global health crises.
